# Epidemiology of acute lower respiratory tract infection hospitalizations in Thai children: A 5‐year national data analysis

**DOI:** 10.1111/irv.12911

**Published:** 2021-09-15

**Authors:** Phanthila Sitthikarnkha, Rattapon Uppala, Sirapoom Niamsanit, Sumitr Sutra, Kaewjai Thepsuthammarat, Leelawadee Techasatian, Jamaree Teeratakulpisarn

**Affiliations:** ^1^ Department of Pediatrics, Faculty of Medicine Khon Kaen University Khon Kaen Thailand; ^2^ Epidemiology Unit, Faculty of Medicine Khon Kaen University Khon Kaen Thailand

**Keywords:** children, hospitalization, lower respiratory tract infection, mortality, pneumonia

## Abstract

**Background:**

Lower respiratory tract infections (LRTIs) are the most common cause for hospitalization in pediatric patients. Pediatric patients with LRTIs are at an increased risk of morbidity and mortality. The national data analysis of epidemiologic variations facilitates awareness and develops solutions to prevent these conditions in the future.

**Objective:**

This study aims to evaluate the epidemiology, causative pathogens, morbidity, and mortality of LRTIs in pediatric patients of Thailand from 2015 to 2019.

**Methods:**

This was a retrospective study among pediatric patients aged between 0 and 18 years old admitted in hospitals due to LRTIs in Thailand from January 2015 to December 2019. The data were extracted from National Health Security Office using the International Statistical Classification of Diseases and Related Health Problems, 10th Revision, Thai Modification; ICD‐10‐TM of J09 to J22.

**Results:**

A total of 1,423,509 children hospitalized due to LRTIs were identified. Most of the patients were of age 1–5 years. Pneumonia was the most common LRTI (876,557 children, 61.58%) in hospitalized children. Respiratory syncytial virus (RSV) is the main etiologic pathogen of bronchiolitis, which presents in approximately 10.86% of all episodes. Influenza viruses were found predominantly in children with pneumonia (15.52%). The mortality rate since 2015–2019 was highest in pneumonia under 1 year old (*P* < 0.001). Pneumonia in children under 5 years old had the highest mortality rate, which accounted for 11.85 per 100,000 children in 2019.

**Conclusions:**

LRTIs had a high incidence rate of hospitalization and mortality, especially in children under 5 years old. Influenza virus was the most common pathogen of pneumonia.

## 1 INTRODUCTION

Lower respiratory tract infections (LRTIs) are a group of infections involving the respiratory tract below the level of the larynx. Globally, the incidences of LRTIs among children under 5 years of age have been observed to be 12,197.8 new cases per 100,000 children.^1^ LRTIs are the leading cause of hospitalizations in the pediatric population.^2^, ^3^ Notably, pneumonia ranks among the top 10 conditions with respect to the cost of hospitalization^3^ and has been found to be 0.22 times per child per year in developing countries.^4^ The Global Burden of Disease Study 2017 reported^1^ that LRTIs caused 808,920 deaths in children younger than 5 years, and no difference in the under 5 LRTIs mortality was observed between the sexes worldwide. In Thailand, Teeratakulpisarn *et al* ^5^ assessed the data of children under 5 years of age from the three national insurance schemes in 2010 and found that 25% of the pediatric patients with LRTIs required hospitalization. The overall mortality of LRTIs in Thai children was 3.96 per 100,000 children, while pneumonia was the most common cause of death.

The pathogens that cause LRTIs vary depending on the patient's age. The respiratory syncytial virus (RSV) and influenza viruses are the common causes of LRTIs in children.^6^ The Global Burden of Disease Study 2017 contributes 11.5% of LRTI episodes due to the influenza virus in all ages.^7^ The seasonal pattern of viruses that cause LRTIs varies depending on the different regions of the world.^8^ In countries of the northern hemisphere, the RSV season usually occurs during winters.^9^ The influenza season also presents during the cold winter months, related to the increased person‐to‐person transmission when indoors and exposure to low absolute humidity.^10^, ^11^ In the tropical region, the influenza cases were detected in different patterns and lacked seasonality variation.^12^ The influenza pattern in tropical Southeast Asia has been less well studied compared with that in industrialized countries. In 2020, Suntronwong *et al* ^13^ evaluated the virology study of influenza depending on the seasonal change and discovered two seasonal waves of high influenza infections, during February and August to September, related to the most humid months of the year. Apart from the seasonal variation of the influenza virus, a study found a correlation between the incidence of pediatric influenza‐associated hospitalization in high‐poverty and high‐crowded areas.^14^

LRTIs exert a significant burden on the patients, families, and budgets of the public healthcare systems. WHO developed the Global Action Plan for Prevention and Control of Pneumonia (GAPP)^15^ in 2009 to increase pneumonia and provide logistical guidance. In Thailand, the Bureau of General Communication Disease, Ministry of Public Health Thailand had adopted and modified the Acute Respiratory Infection in Children Program with the Standard Case Management approach from the WHO recommendations to reduce the morbidity and mortality of pneumonia in children under 5 years of age. Since the WHO recommendations, there is limited information describing the burden and epidemiology of pediatric LRTIs in hospital settings in Thailand. This study aims to evaluate the epidemiology, causative pathogens, morbidity, and mortality of LRTIs in pediatric patients of Thailand from 2015 to 2019.

## 2 MATERIALS AND METHODS

### 2.1 Study design

A retrospective study was conducted on the data of pediatric patients aged under 18 years old who were admitted due to LRTIs in the public hospitals of Thailand from January 2015 to December 2019. The data were extracted from the National Health Security Office using the International Statistical Classification of Diseases and Related Health Problems, 10th Revision, Thai Modification (ICD‐10‐TM). The LRTIs were defined using ICD‐10‐TM as pneumonia (J09–J18), bronchiolitis (J21), and bronchitis (J20). Influenza virus infections that caused LRTIs were classified using ICD‐10‐TM as J09, J10, and J11. For RSV LRTIs, we used ICD‐10‐TM as J12.1, J20.5, and J21.0. Human metapneumovirus (HMPV) was collected using ICD‐10‐TM as J21.1 and J12.3. In addition, the ICD‐10‐TM as J20.6 for rhinovirus, J15.2 for Staphylococcus, and J13 and J15.4 for Streptococcus were also collected for data analysis.

The data collection included the patient's age, gender, month and year of admission, hospital level, hospital region, complication, hospital length of stay (LOS), and discharge status. This study was approved by the institutional review board of the Khon Kaen University, Human Research Ethics Committee (#HE641145).

### 2.2 Statistical analysis

All statistical analyses were performed using the STATA software version 10 (StataCorp LP). The demographic characteristics of the patients were described using the frequency and percentage for categorical data. The continuous data were described as the mean and standard deviation. The admission and mortality rates were calculated based on the universal coverage (UC) scheme. The mortality rate was calculated as per 100,000 population of age groups.

The data of pathogen variation by season each year were presented as monthly trends. The association between the outcome of LRTIs, including the morbidity, mortality, and patient subgroup classifications by age as under 1 year, 1 to under 5 years, and 5 to under 18 years old were calculated using the chi‐square test. The values of *P* < 0.05 were considered to indicate the statistical significance. The 95% confidence interval (CI) of the rate was computed based on the normal approximation to the binomial distribution.

## 3 RESULTS

During the study period, there were 1,423,509 children with LRTIs admitted at the hospitals. It was observed that boys had a slightly higher predominance all year. More than half of the children were aged between 1 and 5 years old. There were 876,557 (61.58%), 397,699 (27.94), and 149,253 (10.48%) patients who contributed to hospitalizations for pneumonia, bronchitis, and bronchiolitis, respectively. Almost all children with bronchiolitis were aged under 5 years (143,515/149,253, 96.16%). The highest hospitalization rates of all LRTIs appeared to geographically cluster within the northeastern region of Thailand (524,071/1,423,509, 36.82%). The majority of children with LRTIs were admitted to secondary‐level hospitals (1,057,067/1,423,509, 74.26%). Table 1 presents the patient characteristics in all age groups divided by the diagnosis of LRTIs.

**TABLE 1:** Patient characteristics

ICD‐10‐TM	J09–J18	J21	J20	Total
Principal diagnosis	Pneumonia (N = 876,557)	Bronchiolitis (N = 149,253)	Bronchitis (N = 397,699)	(N = 1,423,509)
Gender, No. (%)				
Male	505,532 (57.67)	93,502(62.65)	236,087(59.36)	835,121(58.67)
Age group, No. (%)				
<1 year	210,957 (24.07)	55,701 (37.32)	70,015 (17.61)	336,673 (23.65)
1 to <5 years	500,803 (57.13)	87,814 (58.84)	240,773 (60.54)	829,390 (58.26)
5 to <18 years	164,797 (18.80)	5738 (3.84)	86,911 (21.85)	257,446 (18.09)
Region, No. (%)				
Northeast	313,892 (35.81)	75,237 (50.41)	134,942 (33.93)	524,071 (36.82)
South	174,271 (19.88)	25,849 (17.32)	81,174 (20.41)	281,294 (19.76)
Central	170,363 (19.44)	20,943 (14.03)	87,745 (22.06)	279,051 (19.60)
North	74,516 (8.50)	12,316 (8.25)	35,484 (8.92)	122,316 (8.59)
East	54,667 (6.24)	6,367 (4.27)	20,560 (5.17)	81,594 (5.73)
West	44,991 (5.13)	6,126 (4.10)	25,499 (6.41)	76,616 (5.38)
Bangkok	43,857 (5.00)	2,415 (1.62)	12,295 (3.09)	58,567 (4.11)
Level of hospital, No. (%)				
Primary	70,090 (7.99)	10,743 (7.20)	24,426 (6.14)	105,259 (7.39)
Secondary	606,892 (69.24)	124,300 (83.28)	325,875 (81.94)	1,057,067 (74.26)
Tertiary	170,292 (19.43)	12,881 (8.63)	34,407 (8.65)	217,580 (15.29)
Private	29,283 (3.34)	1,329 (0.89)	12,991 (3.27)	43,603 (3.06)

### 3.1 Etiologic pathogen

The primary pathogen that caused LRTIs in Thai children was the influenza virus which accounted for 9.56% (136,074/1,423,509) of all hospitalized children with LRTIs and 6.38% (74,447/1,166,063) of hospitalized children under 5 years with LRTIs. While RSV was the second most common pathogen (16,216/1,423,509, 1.14%), the HMPV was also gradually detected from 2015 to 2019. In addition, staphylococcal and streptococcal LRTIs were seen in 562 and 1197 children, respectively.

**FIGURE 1:**
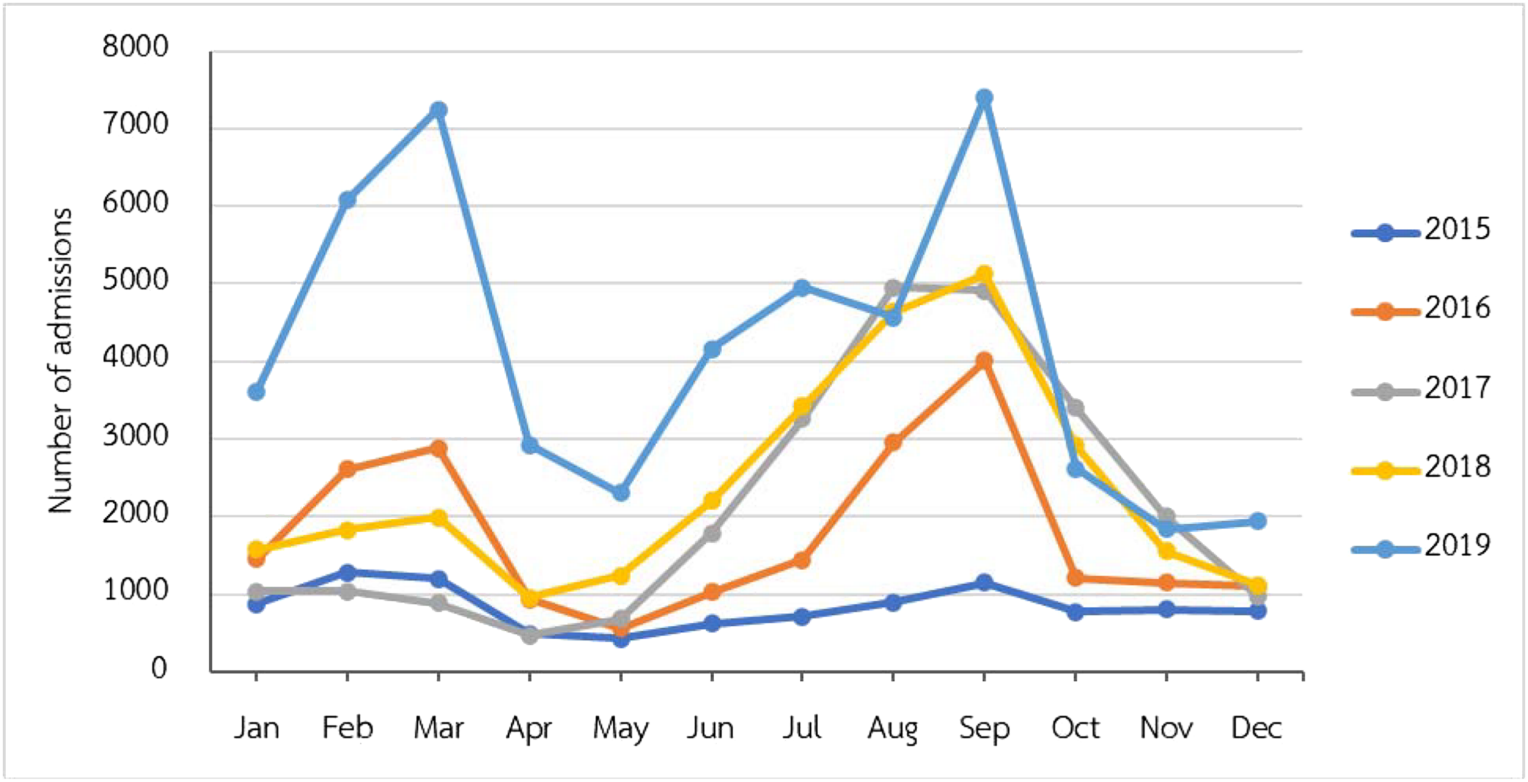
Monthly climate trends of number of admissions in children with influenza lower respiratory tract infections (LRTIs) from 2015 to 2019

**FIGURE 2:**
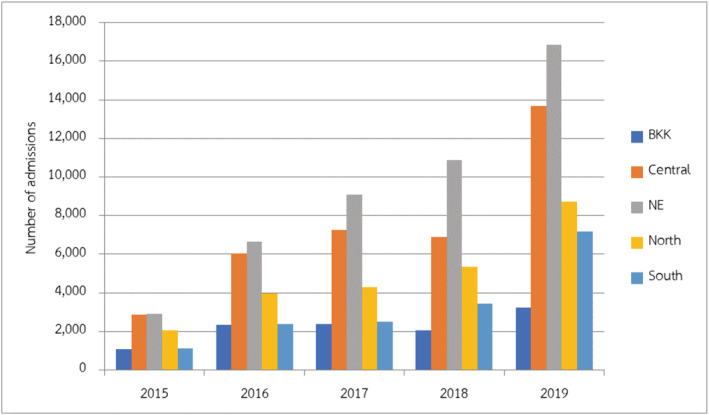
Distribution of number of admissions in children with influenza lower respiratory tract infections (LRTIs) divided by the region of Thailand

A total of 136,074 children had LRTIs from influenza infection. Influenza LRTIs have been increasingly detected every year since 2015, as shown in Figure 1. Children older than 1 year presented with LRTIs caused by influenza 89.59% (121,910/136,074). Two peaks of seasonal distribution of influenza LRTIs were noted during February to March and July to September, which accounted for 55.73% (75,837/136,074) of the total influenza cases. The burden of influenza was highest in the northeastern region of Thailand (46,602/136,074, 34.25%) (Figure 2). The majority of the children with influenza LRTIs were admitted to secondary‐level hospitals (86,875/136,074, 63.84%).

**FIGURE 3:**
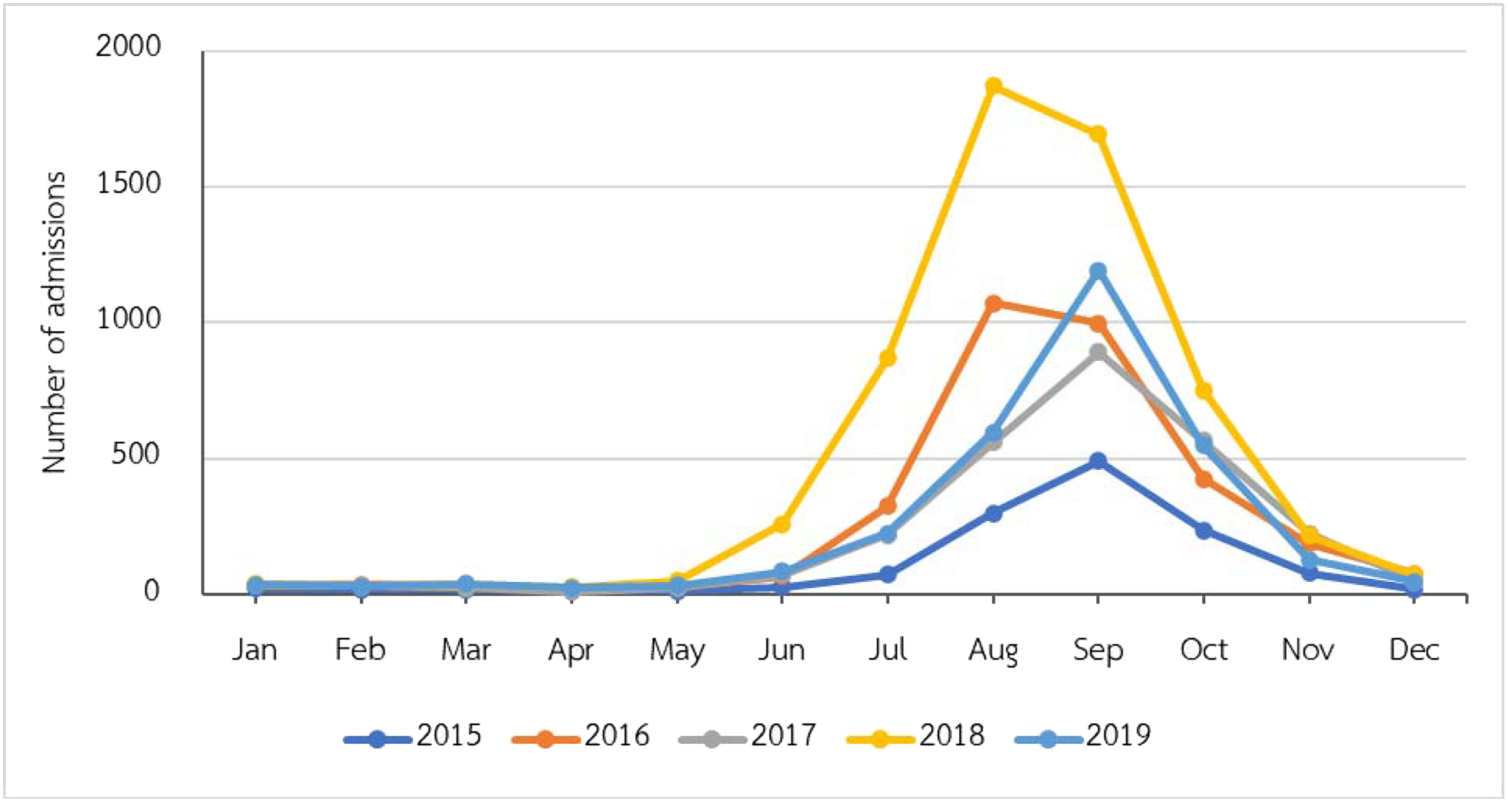
Monthly climate trends of number of admissions in children with respiratory syncytial virus (RSV) lower respiratory tract infections (LRTIs) from 2015 to 2019

**FIGURE 4:**
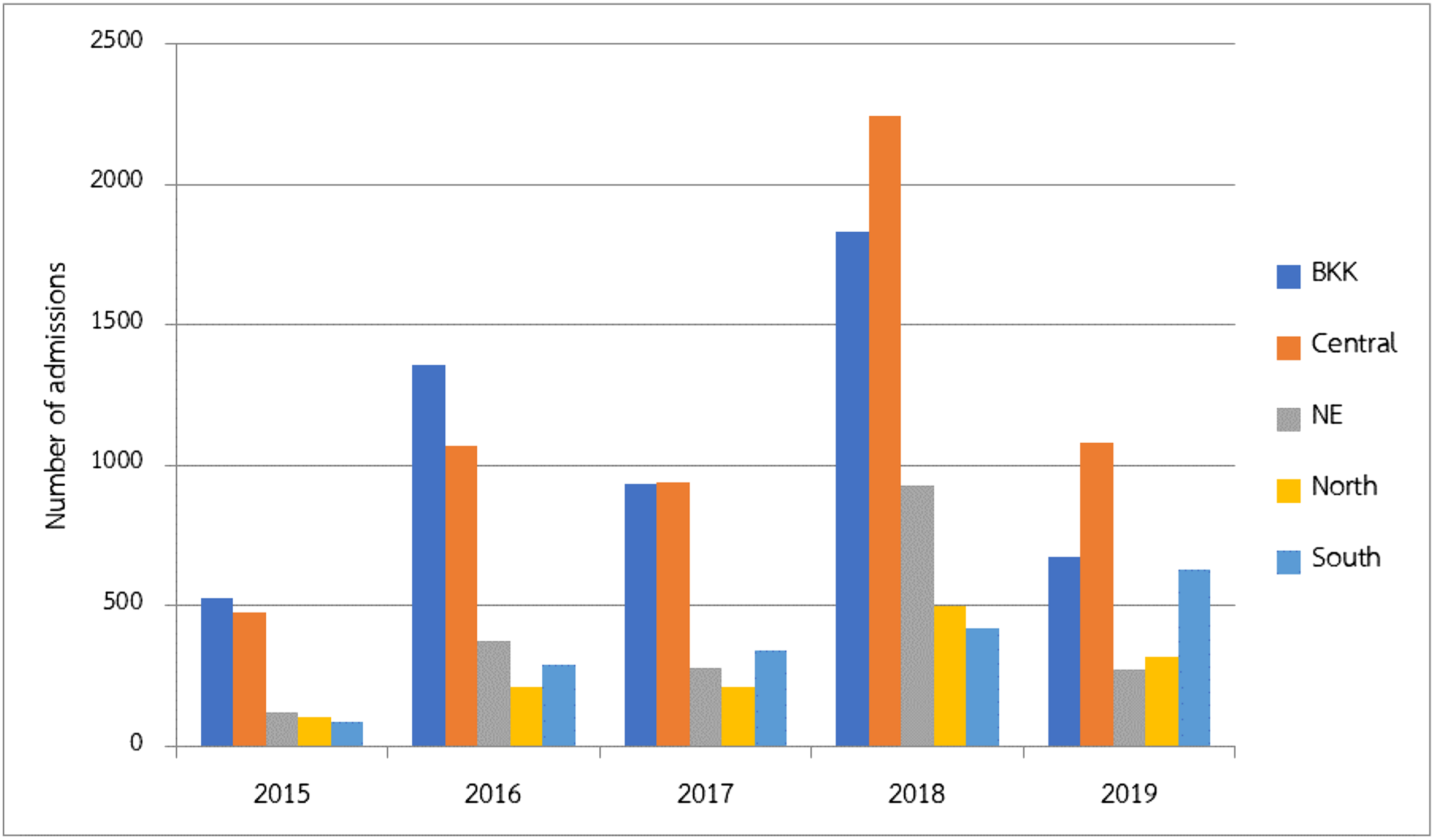
Distribution of number of admissions in children with respiratory syncytial virus (RSV) lower respiratory tract infections (LRTIs) divided by region of Thailand

RSV LRTIs were diagnosed in 16,216 children. It mainly occurred in children under 5 years of age, which accounted for 98.19% of all children with RSV (15,923/16,216). Additionally, it had a high peak incidence during August to October, accounting for 75.14% (12,185/16,216) (Figure 3). Bangkok, the capital city of Thailand, had 5321 children admitted with RSV LRTIs that was 32.81% of all LRTIs (Figure 4). Half of the children were diagnosed in tertiary care hospitals (8734/16,216, 53.86%).

### 3.2 Outcome of treatment

A total of 27,492 children (1.93%) with LRTIs developed respiratory failure that needed endotracheal intubation. It occurred in children with pneumonia accounting for 95.63% (26,290/27,492) of the cases. The respiratory failures due to each etiologic pathogen, such as staphylococcal and streptococcal pneumonia were 46.26% and 29.16% (260/562 and 349/1197) of the cases, respectively, while only 0.45% of influenza pneumonia caused respiratory failure (607/136,074).

The average length of hospital stay for all LRTIs in 2019 was 3.49 days (95% CI 3.48–3.5). The average time taken by pneumonia patients in hospital was 4.05 days (95% CI 3.85–3.89), which was longer than bronchiolitis (3.02 days, 95% CI 3–3.04), and bronchitis (2.72 days, 95% CI 2.71–2.73), respectively. Children aged under 1 year had the most extended average hospital stay due to pneumonia, bronchiolitis, and bronchitis, which accounted for 5.1, 3.3, and 3.1 days, respectively.

**FIGURE 5:**
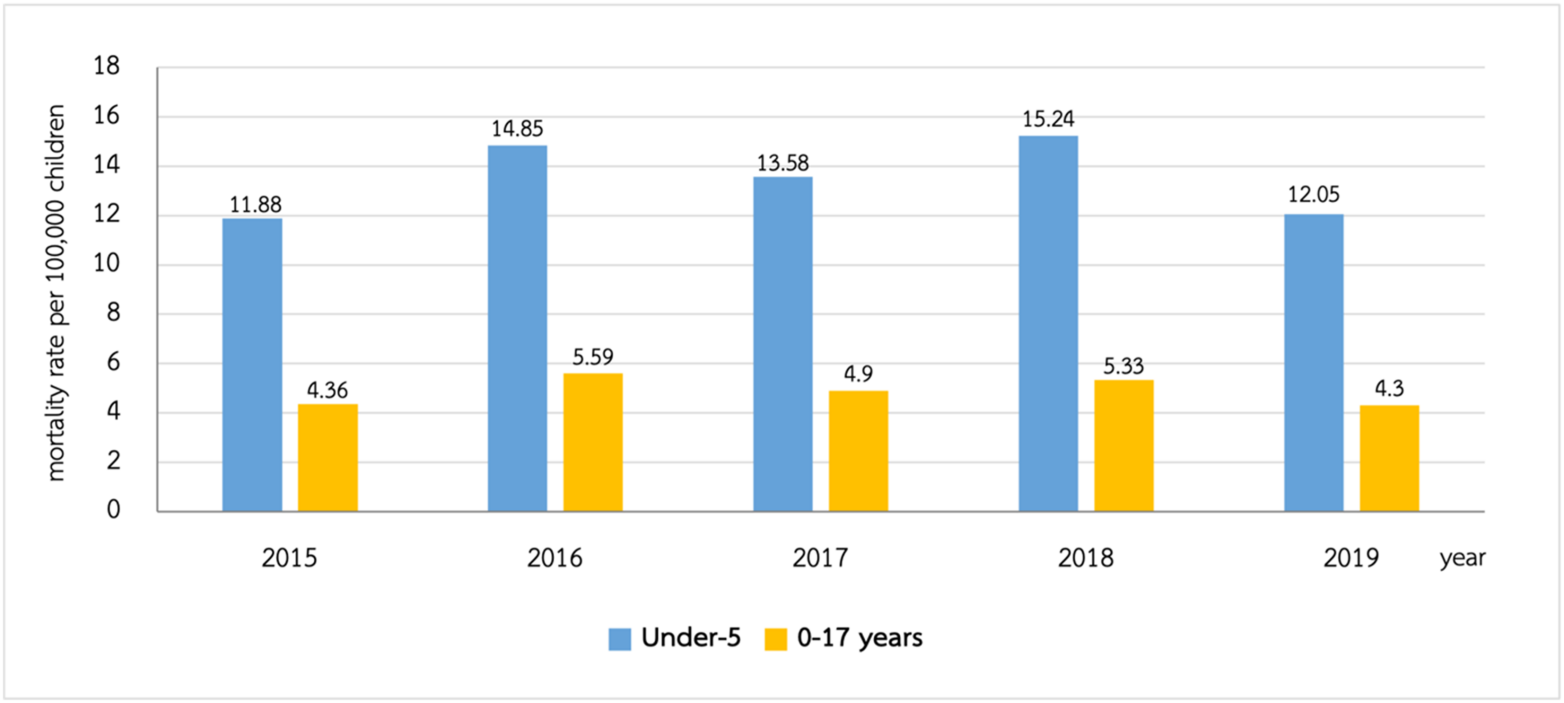
The mortality rate of lower respiratory tract infections (LRTIs) in Thai children during 2015–2019

**FIGURE 6:**
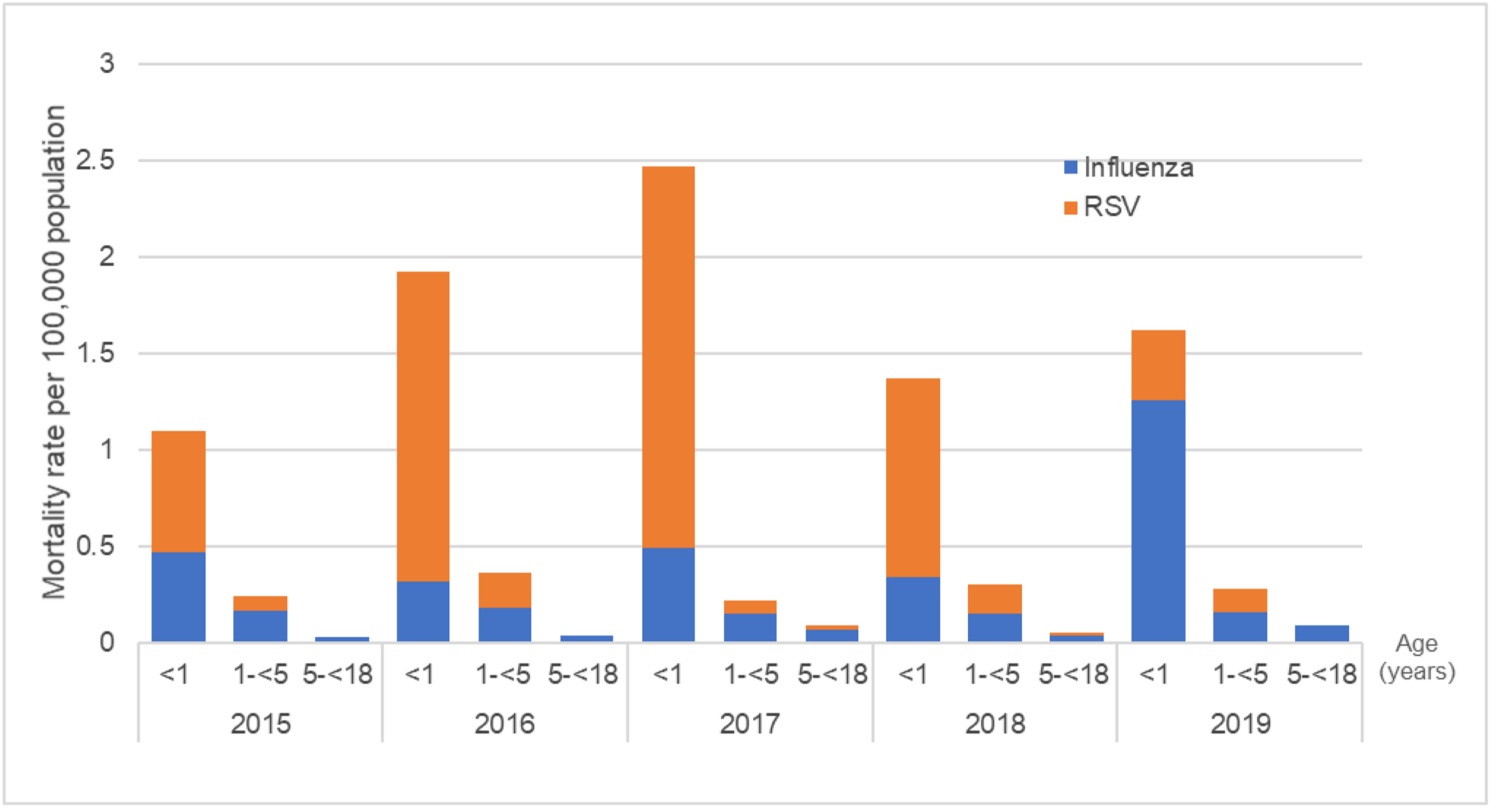
The mortality rate of influenza and respiratory syncytial virus (RSV) associated lower respiratory tract infections (LRTIs) in Thai children during 2015–2019

During the 5‐year study period, LRTIs were responsible for 3168 deaths. The overall mortality of LRTIs during 2015–2019 is shown in Figure 5. Among children in all the age groups, the mortality rate was 4.30 per 100,000 children in 2019. The highest mortality related LRTIs occurred in children under 1 year of age, which accounted for 43.15% (1367/3168). In children under 5 years of age, the mortality rate of LRTIs was 12.5 per 100,000 children per year in 2019. Pneumonia was the most common etiology of LRTI‐related mortality (3120/3168, 98.48%), two thirds of the patients were under 5 years of age. The mortality had a statistically significant occurrence in different age groups among pneumonia in children (Table 2). The etiologic pathogens of LRTIs mortality each year are presented in Figure 6.

**TABLE 2:** Mortality rate of pneumonia in each age group

Age (years)	Death, No. (%)		*P* value
	Yes	No	
<1 year	1347 (43.17)	209,610 (24.00)	<0.001
1 to <5 years	862 (27.63)	499,941 (57.24)	
5 to <18 years	911 (29.20)	163,886 (18.76)	

## 4 DISCUSSION

Thailand has been an upper middle‐income country since July 2011 from the World Bank announcement.^16^ The national health insurance in Thailand contains three different schemes. The Thai government launched a UC scheme in 2002,^17^ which covers most Thai citizens currently (approximately 47.8 million people, 72%).^18^ This study evaluated all pediatric patients under 18 years of age, who were admitted to hospitals based on the UC scheme. Hence, the data covered two thirds of the Thai population.

These data show that LRTIs are still a significant disease burden on the pediatric population in Thailand. Although there was the implementation of the GAPP in 2009 from WHO^15^ and Thailand's national strategic plan for emerging infectious diseases 2017–2021 in 2016,^19^ the burden of LRTIs in Thai children who needed hospitalization in 2010 was similar as 2019, 276,254 and 282,590, respectively.^5^ Pneumonia was also the principal diagnosis of LRTIs during 2015–2019, same as 2010 in Thailand^5^ and other countries in Asia.^20^ The most common causative pathogen of LRTIs was a viral pathogen. There was a rapid test for influenza and RSV in Thailand in secondary and tertiary hospitals. However, the definite causative pathogen is still underreported, especially bacterial pathogens, due to limited resources.

Although the rate of hospitalization due to LRTIs was similar to that in 2010, the overall LRTIs mortality during 2015–2019 was higher than in the past decade, in which the number of deaths of children under the age of 5 years from LRTIs increased from 10.65 per 100,000 population of age group per year in 2010 to 12.05 per 100,000 children per year in 2019. Nevertheless, this study did not identify the risk factors associated with mortality which may represent the cause of a higher mortality rate compared with the previous decade study.^5^

We determined that the influenza virus was the most common cause of LRTIs hospitalizations in children under 5 years of age (6.38%). These results were similar to the previous global study^21^ in 2018 and the study in Vietnam,^22^ the neighboring country of Thailand, that showed the incidence of influenza‐associated LRTIs was 5% and 8.7% of all‐cause LRTIs in children under 5 years of age, respectively. The incidence in the last 5 years was the same as the previous study in Thailand, in which 8% of hospitalized children had been diagnosed with influenza‐associated LRTIs.^23^ Our research found that the influenza LRTIs hospitalization burden increased each year from 2015 to 2019. This may result from additional available confirmed virology tests of influenza in secondary‐level hospitals. Two peaks of seasonal distribution of influenza LRTIs were noted during February to March and July to September, which had a similar pattern as the previous studies in Asia and Thailand.^13^, ^24^ The peak incidence during July to September may be associated with the rainy season in Thailand, similar to the prior study.^25^ Due to the effect of temperature on the seasonal variation of influenza,^10^, ^25^ during February to March, Thailand's winter season could explain the peak incidence of influenza LRTIs. The studies of Suntronwong *et al* ^13^ demonstrated that some subtypes of influenza virus activity were associated with increased humidity.

In this study, the incidence of influenza LRTIs mainly occurred in the northeastern region in Thailand, which has one third of the total Thai population. The population densities in this region were 130.37 people/km^2^ in 2019,^26^ representing the third range in Thailand apart from Bangkok and the central area, respectively. In addition, this region had the lowest average monthly income per household in Thailand,^27^ which represents a low socioeconomic status. Apart from the seasonal variation, these factors may increase the incidence of influenza LRTIs in this region because the low socioeconomic status may have crowded households and decreased ventilation, increasing the person‐to‐person contact rates and risks of viral spread to children via respiratory droplets from adults or individuals from the household.^14^, ^25^

Because children under the age of 5 years with influenza LRTIs had a high incidence of mortality similar to the previous study, the prevention strategies should be concentrated in this group.

Influenza vaccines showed moderate overall protection against influenza‐associated hospitalization and mortality in children who received annual vaccines.^28^, ^29^ Since 2010, Thailand's Advisory Committee on Immunization Practice (ACIP) had implemented the National Influenza Vaccination campaign, which provided free‐of‐charge influenza vaccines to five high‐risk groups. Only healthy children aged 6 months to 2 years of age and children with chronic diseases were included in this campaign.^30^, ^31^ However, only 0.9% of children aged 6 months to 2 years old had received this vaccine in 2012.^31^ Nevertheless, such children benefited from vaccination. The vaccine strategies should be changed to cover children aged 6 months to 5 years of age and implemented before the influenza season begins each year and protecting all children with chronic medical conditions.

The data in this study demonstrated that RSV was the second most common pathogen of LRTIs in the pediatric population in Thailand, especially in children under the age of 5 years. Acute bronchiolitis, which is the disease that occurred in children under 2 years old, was the most common diagnosis of RSV infections, similar to the previous study.^20^ The highest incidence of RSV LRTIs was detected in tertiary care hospitals, mainly in Bangkok, the capital city of Thailand. It may be because of additional rapid RSV antigen tests than in hospitals in the rural area. RSV LRTIs had a high peak incidence during August to October, similar to the previous study in Thailand.^32^ These months were stated as the rainy season of Thailand. Several studies in tropical and developing countries presented the association between rain and RSV infection.^25^

Although some studies found that RSV LRTIs had a higher severity of disease than other pathogens,^33^, ^34^ our study showed a lower mortality of RSV LRTIs than the previous study. The reason could be the unavailability of investigations to determine the causative organism in certain areas of Thailand. Currently, RSV vaccines are available globally. Although palivizumab is effective for RSV infections, it is not yet available in Thailand. Therefore, infection prevention and control strategies are recommended to reduce the transmission of RSV infections. WHO and CDC recommended that this virus is mostly transmitted by the contact and droplet route.^11^ Some studies found that contact precaution can prevent RSV transmission in healthcare settings.^35^ Because contact transmission is the primary route of transmission of RSV, the pediatrician should educate the parents and caregivers about the methods that can prevent children from contracting infections, such as adequate hand washing. This method can significantly reduce the rate of RSV infections from 4.2% to 0.6%.^36^

Several limitations need consideration in our study. First, this was a retrospective study that may have some missing data. Second, the estimated burden, morbidity, and mortality of LRTIs in this study are limited by the data availability. The data in this study were extracted from ICD‐10‐TM codes that can be miscoded. Third, the etiology of LRTIs may be underdetected due to the high cost of PCR and the difficulty to obtain a specimen for culture in children, especially in primary‐level hospitals. Therefore, the majority of the diagnoses in this study were unspecified for etiology pathogens. Finally, the risk factors associated with mortality were not evaluated in this study. Hence, we cannot conclude the reason for higher mortality in this study.

## 5 CONCLUSIONS

LRTIs had a high incidence rate of hospitalization in children. The influenza virus was the most common pathogen of pneumonia. The mortality rate mainly occurred in children with LRTIs aged under 5. Therefore, the public intervention to prevent LRTIs in children should be done before spreading viral infections during the spreading season.

## AUTHOR CONTRIBUTIONS

**Phanthila Sitthikarnkha:** Conceptualization; data curation; formal analysis; investigation; methodology; resources; validation; visualization. **Rattapon Uppala:** Conceptualization; data curation; formal analysis; funding acquisition; investigation; methodology; project administration; resources; supervision; validation; visualization. **Sirapoom Niamsanit:** Conceptualization; validation. **Sumitr Sutra:** Conceptualization; data curation; formal analysis; methodology; resources; supervision; validation. **Kaewjai Thepsuthammarat:** Data curation; formal analysis; resources; software; validation. **Leelawadee Techasatian:** Conceptualization; methodology; supervision. **Jamaree Teeratakulpisarn:** Methodology; supervision.

## CONSENT FOR PUBLICATION

The author signs for and accepts responsibility for releasing this material on behalf of any and all coauthors.

### PEER REVIEW

The peer review history for this article is available at https://publons.com/publon/10.1111/irv.12911.

## Data Availability

The datasets generated and/or analyzed during the current study are not publicly available but available from the corresponding author (RU) on request.
